# Oral vitamin A supplementation of porcine epidemic diarrhea virus infected gilts enhances IgA and lactogenic immune protection of nursing piglets

**DOI:** 10.1186/s13567-019-0719-y

**Published:** 2019-11-29

**Authors:** Stephanie N. Langel, Francine Chimelo Paim, Moyasar A. Alhamo, Kelly M. Lager, Anastasia N. Vlasova, Linda J. Saif

**Affiliations:** 1 Food Animal Health Research Program, Ohio Agricultural Research and Development Center, College of Food, Agriculture and Environmental Sciences, Department of Veterinary Preventive Medicine, College of Veterinary Medicine, The Ohio State University, Wooster, OH USA; 2 National Animal Disease Center, Agricultural Research Service, USDA, Ames, IA USA

## Abstract

Vitamin A (VA) has pleiotropic effects on the immune system and is critical for mucosal immune function and intestinal lymphocyte trafficking. We hypothesized that oral VA supplementation of porcine epidemic diarrhea virus (PEDV)-infected pregnant gilts would enhance the gut-mammary gland-secretory IgA axis to boost lactogenic immunity and passive protection of nursing piglets against PEDV challenge. Gilts received daily oral retinyl acetate (30 000 IU) starting at gestation day 76 throughout lactation. At 3–4 weeks pre-partum, VA-supplemented (PEDV + VA) and non-supplemented (PEDV) gilts were PEDV or mock inoculated (mock + VA and mock, respectively). PEDV + VA gilts had decreased mean PEDV RNA shedding titers and diarrhea scores. To determine if lactogenic immunity correlated with protection, all piglets were PEDV-challenged at 3–5 days post-partum. The survival rate of PEDV + VA litters was 74.2% compared with 55.9% in PEDV litters. Mock and mock + VA litter survival rates were 5.7% and 8.3%, respectively. PEDV + VA gilts had increased PEDV IgA antibody secreting cells and PEDV IgA antibodies in serum pre-partum and IgA^+^β7^+^ (gut homing) cells in milk post piglet challenge compared with PEDV gilts. Our findings suggest that oral VA supplementation may act as an adjuvant during pregnancy, enhancing maternal IgA and lactogenic immune protection in nursing piglets.

## Introduction

Diarrheal disease represents a major global health burden and is the leading cause of morbidity and mortality in young humans and animals alike [1, 2]. In young animals, diarrheal disease accounts for an estimated multi-million dollar loss annually to the livestock industry due to mortality, reduced weight gain, treatment costs and trade sanctions on exporting animal products from infected countries [3, 4]. Passive lactogenic immunity that provides protective IgG in colostrum and a continuous supply of secretory IgA (sIgA) in milk plays a significant role in conferring protection against enteric pathogens in neonates [5]. For example higher pathogen-specific IgA antibody (Ab) titers in milk are associated with lower incidence of enteric disease in pigs and children [6–9]. Additionally, boosting the maternal immune response may have beneficial consequences for the mother, providing protection against disease during pregnancy and lactation. Enhancing immunity by maternal vaccination has the potential to provide a dual benefit in immune protection for the mother–neonatal dyad.

Porcine epidemic diarrhea virus (PEDV) is an alphacoronavirus that causes acute diarrhea, dehydration and 80–100% mortality in neonatal piglets [2, 10]. In adult pigs, PEDV causes watery diarrhea, depression and anorexia as well as agalactia and reduced reproductive performance [2, 11]. Due to the high virulence of PEDV and the naïve, immature immune system of neonatal piglets, passive lactogenic immunity to PEDV induced via the gut-mammary gland (MG)-sIgA axis during pregnancy and lactation remains the most promising and effective way to protect nursing piglets against PEDV-induced disease [8, 12–15]. Providing sufficient PEDV-specific lactogenic immunity is dependent on trafficking of IgA antibody secreting cells (ASCs) from the intestine to the MG and accumulation of sIgA Abs in milk [7, 8, 13, 14, 16]. However, little is known regarding the anti-viral humoral immune response during pregnancy and the level of lactogenic immune protection generated after induction of the gut-MG-sIgA axis is variable. Indeed, our lab demonstrated that third trimester PEDV-infected gilts provided insufficient passive lactogenic immune protection to PEDV-challenged nursing piglets compared with second or first trimester PEDV-infected gilts [16]. Therefore, identifying cost effective strategies to stimulate and enhance trafficking of PEDV IgA ASCs to the MG and accumulation of PEDV sIgA Abs in milk in a pregnant swine model is critical for development of maternal vaccines and therapeutics that improve maternal and lactogenic immunity and neonatal health.

Retinoic acid (RA) is a vitamin A (VA) metabolite with pleiotropic effects on the immune system [17, 18]. For example, VA deficient (VAD) individuals with serum retinol levels at 0.70 µmol/L or below are more susceptible to some enteric and respiratory diseases, with young children at the highest risk [19, 20]. Specifically, VA is required for the enhancement and regulation of immune responses and cellular trafficking in the gut. *Lamina propria* (LP) CD103^+^ dendritic cells (DCs) synthesize RA through expression of retinal dehydrogenases (i.e. *Aldh1a1/2*) [21]. Synergistically with locally produced cytokines, CD103^+^ LP DCs upregulate mucosal trafficking adhesion molecules, integrin α4β7 and chemokine CC receptor (CCR)9 expression on B and T lymphocytes after interaction in the Peyer’s Patches or mesenteric lymph node (MLN) [22–24]. For example, VAD mice had significantly decreased α4β7^+^ memory T cells in lymphoid organs and T cells in the LP [24]. Additionally, VAD alters cytokine responses during infection by skewing towards an increased T-helper cell type-1 (Th1) cytokine response in infants and mice [25, 26]. Treatment of T cells with 10 nM RA in vitro resulted in a shift toward Th2 cytokine responses [26] promoting an immunoregulatory environment. Therefore, even subclinical deficiencies in VA may negatively impact mucosal lymphocyte trafficking and gut homeostasis during pregnancy.

Also relevant to mucosal immune responses is the role of RA in B cell differentiation and IgA production. Using a gnotobiotic pig model, our lab demonstrated that subclinical VAD piglets had decreased frequencies of CD103^+^ LP DCs, resulting in decreased adaptive immune responses to human rotavirus (HRV) and prolonged diarrhea [27, 28]. VAD gnotobiotic piglets vaccinated with attenuated HRV demonstrated higher diarrhea severity post HRV challenge compared with vaccinated VA sufficient piglets. This coincided with lower serum IgA HRV Ab titers and significantly decreased intestinal IgA ASCs post-challenge suggesting a compromised anamnestic immune response [28, 29]. However, these parameters and the impact of VA supplementation during pregnancy on the gut-MG-sIgA axis and passive neonatal immunity are largely undefined. Therefore, we hypothesized that oral VA supplementation of PEDV-infected pregnant gilts would enhance trafficking of IgA ASCs from the gut to the MG leading to increased accumulation of sIgA Abs in milk and passive lactogenic protection of PEDV-challenged nursing piglets.

The risk of subclinical and clinical VAD increases with gestational age due to accelerated growth of the fetus and the physiological increase of blood volume in swine and humans [27, 30]. Dietary VA recommendations given for swine [31] are represented by physiologically-based mathematical models developed for relatively disease-free environments. Therefore, it is likely that the dietary VA required during pregnancy to efficiently stimulate the gut-MG-sIgA axis and provide sufficient lactogenic immune protection to neonates during an enteric viral infection is higher than current dietary recommendations. To investigate the impact of daily oral VA supplementation during gestation and lactation, we supplemented gilts with non-teratogenic, yet physiologically relevant levels of oral retinyl acetate [30 000 international units (IU)] daily starting at the beginning of the third trimester and lasting throughout lactation. We demonstrated that daily oral VA supplementation in PEDV-infected gilts decreased diarrhea, increased the numbers of PEDV IgA ASCs and PEDV IgA Abs in serum pre-partum and IgA immunoglobulin secreting cells (IgSCs) and IgA^+^β7^+^ cells in milk post piglet challenge, providing greater lactogenic immune protection to neonatal nursing piglets.

## Materials and methods

### Virus

The wild-type PC22A strain of PEDV was used for gilt infection and piglet challenge at a dose of 1 × 10^5^ plaque forming units diluted in Minimal Essential Media [MEM (Life Technologies, Carlsbad, CA, USA)]. Briefly, PC22A was isolated and cultured in Vero cells as described previously [32, 33]. Cells were grown in growth medium containing Dulbecco’s Modified Eagle’s Medium [DMEM (Life Technologies, Carlsbad, CA, USA)] supplemented with 5% fetal bovine serum (Life Technologies, Carlsbad, CA, USA) and 1% antibiotic–antimycotic (Life Technologies, Carlsbad, CA, USA). Virus was grown in Vero cells in maintenance medium containing DMEM supplemented with 10 μg/mL trypsin (Life Technologies, Carlsbad, CA), 0.3% tryptose phosphate broth (Sigma Aldrich, St. Louis, MO, USA), and 1% antibiotic–antimycotic. Cells were kept in a humidified incubator at 37 °C and 5% CO_2_. PC22A was passaged three times in Vero cells before passaging once for generation of inoculum in a gnotobiotic pig. The virulence of pig passaged PC22A was confirmed in adult and neonatal pigs as described previously [13, 34, 35]. Cell-culture adapted PC22A was used as a positive control in the virus neutralization (VN) Ab assay.

### Experimental design

**Figure 1:**
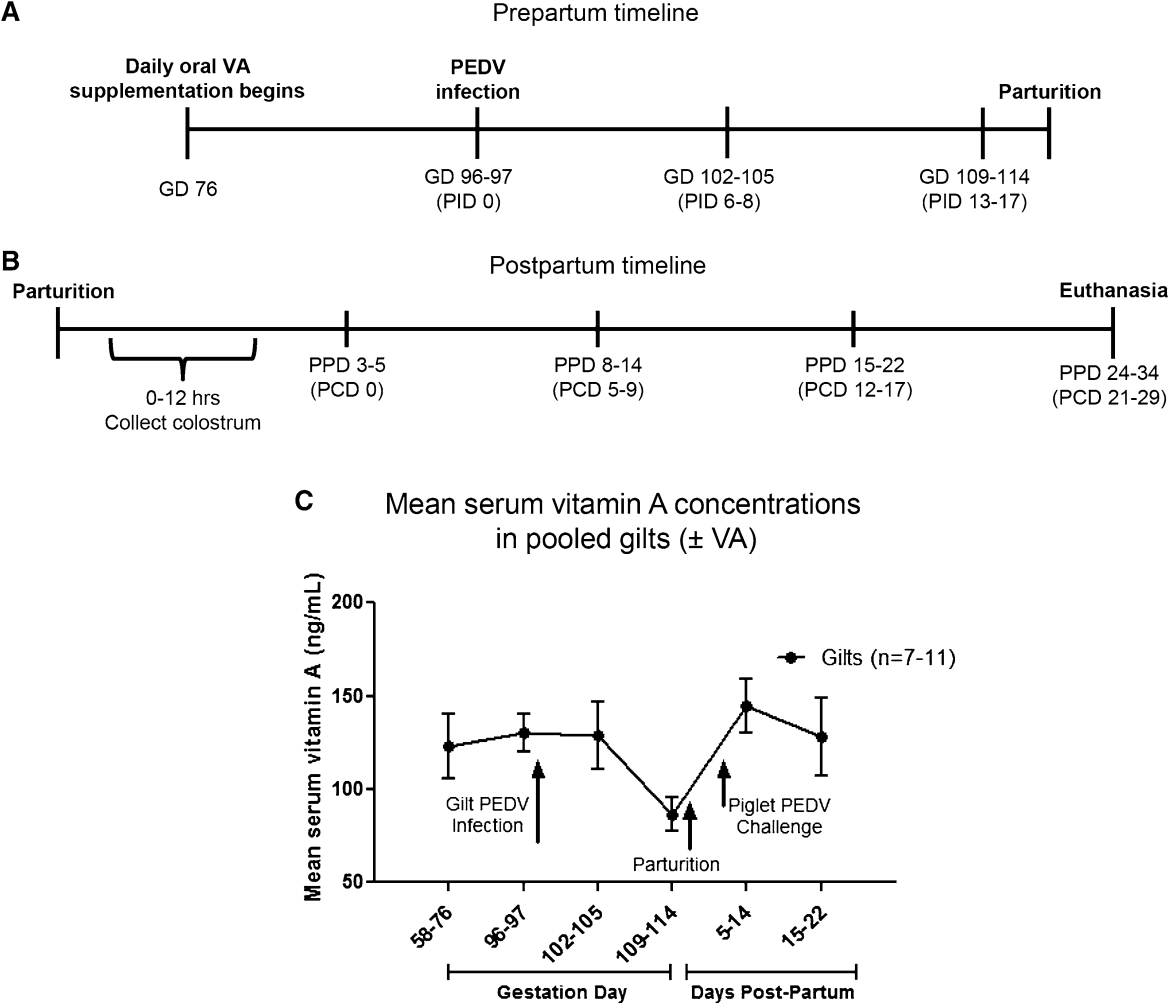
**Serum VA concentrations decreased immediately prior to parturition in gestating gilts. A** Schematic diagram of the experimental design showing gilt PEDV infection and sample time points at post-infection day (PID) 0 [gestation day (GD) 96–97], 6–8 (GD 102–105) and 13–17 (GD 109–114) and **B** piglet PEDV challenge at 3–5 post-partum day (PPD) and sample time points at PPD 3–5 [PCD (post-challenge day) 0], PPD 8–14 (PCD 5–9), PPD 15–22 (PCD 12–17) and PPD 24–34 (PCD 21–29). **C** Pooled serum VA concentrations (ng/mL) in gilts pre and post-partum. Statistical analysis was performed using the two-way ANOVA with repeated measures and Bonferroni’s correction for multiple comparisons. Data are mean ± SEM.

All animal experiments were approved by the Institutional Animal Care and Use Committee at The Ohio State University. All methods were carried out in accordance with approved protocol (2015A00000071) and relevant regulations and pigs were maintained, sampled, and euthanized humanely. First parity PEDV and transmissible gastroenteritis virus (TGEV) seronegative pregnant gilts (Landrace × Yorkshire × Duroc cross-bred) arrived at our facilities from either The Ohio State University swine center facility or a commercial swine herd by gestation day (GD) 70 and housed individually in an open pen during gestation and the majority of lactation. Gilts were placed in farrowing crates 3–5 days prior to expected farrowing and 3–5 days post-partum to ensure safe delivery and interaction with piglets. Artificial light was regulated by a 12:12 light/dark timer. However, natural light also entered the animal rooms from nearby windows. Gilts were randomly assigned to one of four treatment groups: (1) MEM-infected (mock) (*n* = 4); (2) MEM-infected + daily oral VA supplemented (mock + VA) (*n* = 4); (3) third trimester [GD 96–97 (~18 days pre-partum)] PEDV-infected (PEDV) (*n* = 6); and (4) third trimester PEDV-infected + daily oral VA supplemented (PEDV + VA) (*n* = 5). VA-supplemented gilts orally received 30 000 IU/day of retinyl acetate from GD 76 throughout lactation (Figure 1A). Considering the circadian nature of trafficking lymphocytes [36, 37] retinyl acetate was supplemented two times per day (15 000 IU at 0900 h and 15 000 IU at 1700 h). Gilt fecal samples were collected, and clinical signs observed on post-PEDV infection day (PID) 0–15. Fecal consistency was scored as follows: 0, solid; 1, pasty; 2, semi-liquid; 3, liquid, respectively. A fecal consistency score of > 1 was considered as diarrhea [13, 38]. Blood samples were taken on PID 0, 6–8 and 13–17 for serum and mononuclear cell (MNC) isolation (Figure 1A). All gilts naturally farrowed in our facilities at GD 114 (± 3) and colostrum was collected within 12 h of parturition. All piglets were allowed to suckle naturally after birth and were kept with their mothers throughout lactation. Piglets were orally PEDV-challenged at 3–5 days of age (Figure 1B). Gilt and piglet serum was collected on post piglet challenge day (PCD) 0, 5–9, 12–17 and 21–29. All colostrum and milk samples were collected after administration of 2 cc oxytocin intramuscularly (IM) at post-partum day (PPD) 0, 3–5, 8–14 and 15–22 (Figure 1B). Gilt MG biopsies were collected at GD 104–114 and PPD 8–14. Piglet fecal samples were collected, and clinical signs and body weights recorded daily on PCD 0–7 and every other day through PCD 15. All animals were euthanized at PCD 21–29. Gilt blood, ileum, MG, MLN and spleen tissues were collected for MNC isolation. Piglet blood was collected, and the serum separated for immunologic assays.

### Serum VA concentrations

Serum samples were collected as reported previously [27] and submitted to the Diagnostic Center for Population and Animal Health (Michigan State University, Lansing, MI, USA) for quantitative VA concentrations by HPLC.

### PEDV RNA quantification by real-time quantitative polymerase chain reaction (RT-qPCR)

To determine PEDV RNA shedding titers, two rectal swabs were suspended in 4 mL MEM as described previously [35]. Viral RNA was extracted from 50 μL of fecal supernatants following centrifugation (2000 × *g* for 30 min at 4 °C) using the MagMAX Viral RNA Isolation Kit (Applied Biosystems, Foster City, CA, USA) according to the manufacturer’s instructions. Titers of viral RNA shed in feces were determined by TaqMan RT-qPCR using the Onestep RT-PCR Kit (QIAGEN, Valencia, CA, USA) as described previously [35]. The detection limit was 10 copies per 20 µL of reaction, corresponding to 4.8 log_10_ copies/mL of original fecal samples.

### Isolation of MNCs from blood, spleen, MG, MLN and ileum

Blood, spleen, MG, MLN and ileum were collected aseptically at euthanasia and processed for MNC isolation as described previously [16, 39]. The isolated cells were resuspended in enriched RPMI [E-RPMI (Roswell Park Memorial Institute)] medium containing 8% fetal bovine serum, 2 mM l-glutamine, 1 mM sodium pyruvate, 0.1 mM nonessential amino acids, 20 mM HEPES (*N*-2-hydroxy-ethylpiperazine-*N*-2-ethanesulfonic acid), and 1% antibiotic–antimycotic (Life Technologies, Carlsbad, CA, USA) and used for assays. The viability of MNCs was determined by trypan blue exclusion. Briefly, MNCs were diluted twofold in 0.4% trypan blue before visualizing using an automated cell counter (Cellometer, Nexcelom, Lawrence, MA, USA). The viability (%) was calculated as [1.00 − (number of blue cells/number of total cells)] × 100.

### Detection of cytokines in serum by ELISA

Serum samples from PEDV and PEDV + VA gilts were processed and analyzed for proinflammatory [tumor necrosis factor (TNF)-α, interleukin (IL)-6], innate [interferon (IFN)-α] and Th1 (IL-12, IFN-γ), Th2 (IL-4), Th17 (IL-22) and T regulatory/anti-inflammatory [IL-10 and transforming growth factor (TGF)-β] cytokines as described previously [16].

### Colostrum/milk processing for whey and isolation of MNCs

Colostrum/milk was collected aseptically after gilts were given 2 cc oxytocin (VetOne, Boise, ID, USA) IM to facilitate collection of mammary secretions. Samples were filtered through a 70 μm pore filter and centrifuged at 1800 × *g* for 30 min at 4 °C to separate fat, skim milk and cell pellet portions. Fat was removed utilizing sterile plain-tipped applicators (Fisher Scientific, Hampton, NH, USA). Skim milk was collected and centrifuged at 28 000 × *g* for 1 h at 4 °C to separate the whey that was then stored at −20 °C until tested. Colostrum and milk MNCs were isolated as described previously [16]. The viability of MNCs was determined by trypan blue exclusion.

### PEDV plaque reduction VN assay

A plaque reduction VN assay was performed as described previously [16]. Plaques were counted and the VN titers were determined by taking the reciprocal of the highest dilution of a serum/whey sample showing an 80% reduction in the number of plaques compared with seronegative control serum/whey. Samples negative at a dilution of 1:16 were assigned a titer of 1:2 for the calculation of geometric mean titers (GMTs).

### PEDV whole virus Ab ELISA

A PEDV whole virus Ab ELISA was performed as described previously [16]. The ELISA Ab titer was expressed as the reciprocal of the highest dilution that had a corrected *A*_450_ value (sample absorbance in the virus-coated well minus sample absorbance in the mock antigen-coated well) greater than the cut-off value (mean corrected *A*_450_ value of negative controls plus 3 standard deviations). Samples negative at a dilution of 1:4 were assigned a titer of 1:2 for the calculation of GMTs.

### Total and PEDV Enzyme-Linked Immunosorbent Spot (ELISPOT)

Enumeration of total IgSCs and PEDV-specific ASCs was performed as described previously [16, 39]. Counts were averaged from duplicate wells and expressed relative to 5 × 10^5^ MNC.

### Histologic analysis and evaluation of PEDV Ab^+^ cells in the MG

PEDV Ab^+^ cells in the MG were analyzed as described previously [16]. Microscopic images (300× magnification) were obtained using a fluorescence microscope (Olympus IX70-S1F2). Mean numbers of PEDV Ab^+^ cells were evaluated by measuring at least 3–6 different tissue areas at 300× magnification for each sample time point (GD 104–114 and PCD 5–9) from mock, mock + VA, PEDV and PEDV + VA gilts.

### Flow cytometry to assess B/T lymphocytes and homing marker integrin and receptor frequencies

Procedures for flow cytometry staining (including buffers used) were performed as described previously with minor modifications [16, 27]. Briefly, 100 μL of MNCs at 1 × 10^7^ cells/mL were stained with anti-porcine CD21-PE (clone BB6-11C9.6, Southern Biotech, 1:50) and anti-porcine CD2 (clone MSA4, VMRD, 1:50) monoclonal Abs to determine B cell subsets [40]. To determine expression of α4 integrin and β7 integrin, cells were stained with porcine cross-reactive anti-human α4 integrin (clone HP2/1, Abcam, Cambridge, MA, 1:100) and anti-mouse β7 integrin (clone FIB27, BD Biosciences, 1:50) monoclonal Abs. Additionally, to determine expression of IgA, cells were stained with anti-porcine IgA (clone K61 1B4, Bio-Rad, 1:50) monoclonal Ab. After washing, cells were stained with appropriate secondary antibodies. For intracellular CD79β, stained cells were permeabilized with Cytofix/Cytoperm (BD Biosciences), washed with Perm/Wash Buffer (BD Biosciences) and stained with porcine cross-reactive anti-mouse CD79β-FITC Ab (clone AT1072, Bio-Rad, 1:50) monoclonal Ab. Additionally, CD4^+^ (anti-porcine CD4, clone 74-12-4, Southern Biotech, 1:50) and CD8^+^ (anti-porcine CD8, clone 76-2-11, Southern Biotech, 1:50) T cells were assessed within the CD3^+^ (anti-porcine CD3, clone PPT3, Southern Biotech, 1:100) MNC population (T lymphocytes). Appropriate isotype matched control Abs were included. Acquisition of 50 000 events and analyses were done using the Accuri C6 flow cytometer (BD Biosciences, San Jose, CA, USA). All cells were first gated for singlets (FSC-H vs. FSC-A) and MNCs (SSC-A vs. FSC-A). To determine B cell subsets, MNC populations were analyzed by CD79β expression. α4^+^β7^+^ and IgA^+^β7^+^ MNCs were determined within the CD79β^+^ population as described previously [16].

### Statistics

Two-way analysis of variance (ANOVA-general linear model), followed by Bonferroni’s post-test, was used to compare serum VA concentrations, PEDV RNA shedding titers, fecal consistency scores, mean concentrations of serum cytokines, frequencies of blood MNC populations in flow cytometry, PEDV IgA and IgG ASCs, log-transformed PEDV IgA, IgG and VN Ab titers, normalized weights and PEDV Ab^+^ cells in the MG. The frequencies of cell populations and PEDV IgA and IgG ASCs in milk and ileum were compared among groups with the Mann–Whitney (nonparametric) *t* test. The log-rank (Mantel-Cox) test was used for comparison of survival curves amongst treatment groups. Statistical significance was assessed at *P* ≤ 0.05 for all comparisons. Assays were run by investigators blinded to sample and treatment identification prior to analysis. All statistical analyses and random number generation for treatment randomization were performed with GraphPad Prism 5 (GraphPad Software, Inc., CA, USA).

## Results

### Serum VA concentrations decreased in gilts regardless of treatment, immediately prior to parturition

Oral VA supplementation in third trimester gilts did not significantly alter mean serum VA concentrations. However, when gilts (± VA) were pooled, there was a trend for decreased mean serum VA concentrations immediately prior to parturition (Figure 1C).

### PEDV RNA shedding titers and PEDV-induced diarrhea were lower in PEDV + VA gilts

**Figure 2:**
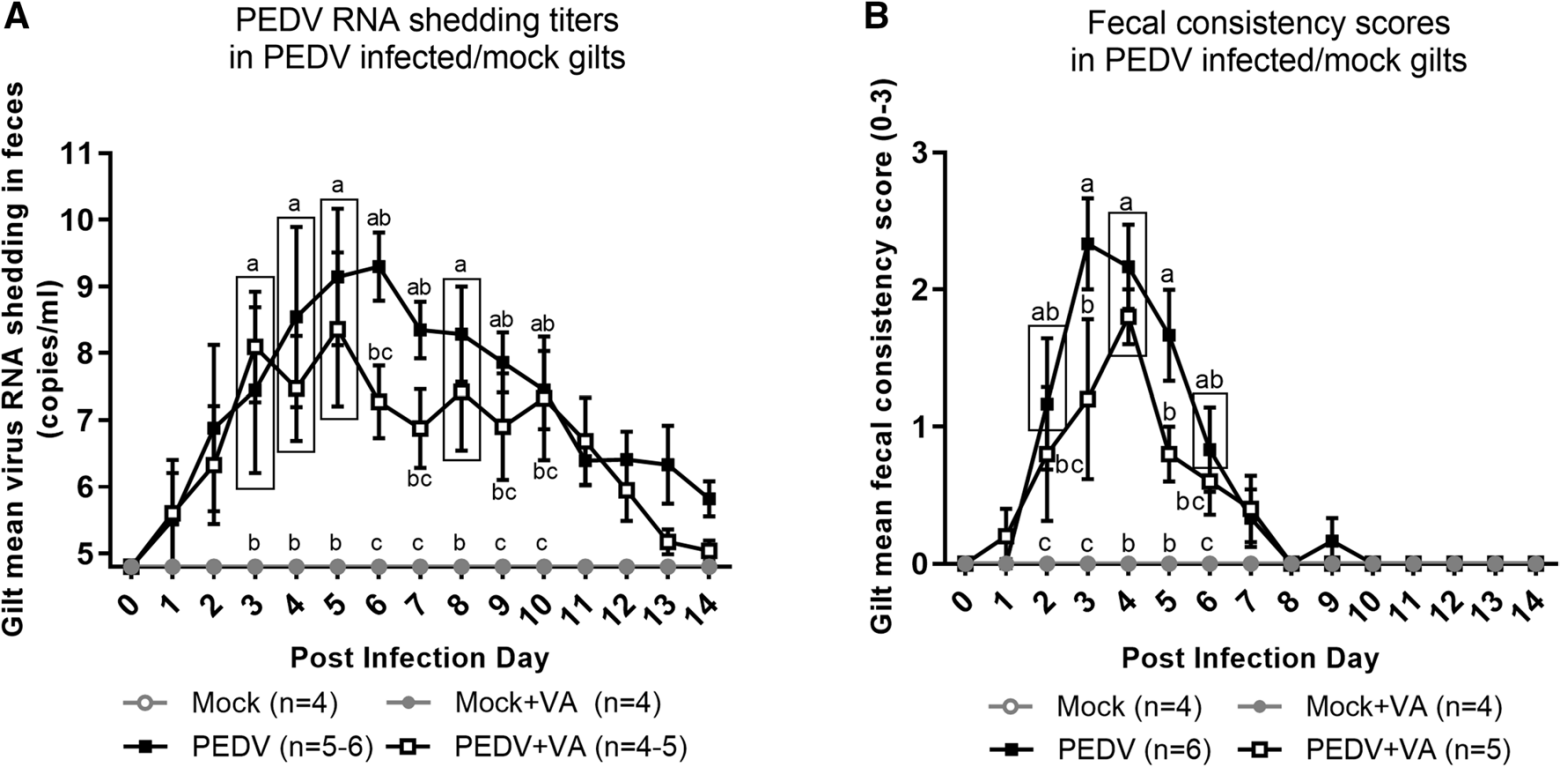
**Daily oral VA supplementation significantly decreased diarrhea in PEDV infected gilts. A** Virus shedding was determined by real-time quantitative polymerase chain reaction (RT-qPCR) and expressed as log_10_ copies/mL. **B** Diarrhea was determined by fecal consistency score > 1 (fecal consistency was scored as follows: 0, normal; 1, pasty/semiliquid; 2, liquid; 3, watery). Different alphabetical letters indicate significant differences among treatment groups (mean ± SEM). Statistical analysis was performed using the two-way ANOVA with repeated measures and Bonferroni’s correction for multiple comparisons. *P < 0.05.

Mean PEDV RNA shedding titers were consistently numerically lower (PID 4–9) and there was a more rapid rate of decrease in mean PEDV RNA shedding titers at PID 12–14 in PEDV + VA compared with PEDV gilts (Figure 2A). Additionally, mean fecal consistency scores at PID 3 and 5 were significantly lower in PEDV + VA compared with PEDV gilts (Figure 2B).

### Oral VA supplementation modulated cytokine immune parameters during PEDV infection

There was a trend for higher IFN-α and IFN-γ, type I and II interferons, respectively, at PID 13–17 in PEDV but not PEDV + VA gilts while immunoregulatory cytokine IL-10 increased numerically at PID 6–8 in PEDV + VA compared with PEDV gilts (Additional file 1). Additionally, there was a trend for increased mean concentrations of IL-22, a cytokine that promotes tissue regeneration and repair in the gut [41] at PID 6–8 in PEDV + VA compared with PEDV gilts (Additional file 1). No other trends were observed for serum concentrations of TNF-α, IL-6, IL-12, IL-4 or TGF-β (Additional file 1).

### Oral VA significantly increased pre-partum levels of circulating PEDV IgA ASCs and Abs and IgA^+^β7^+^ MNCs

**Figure 3:**
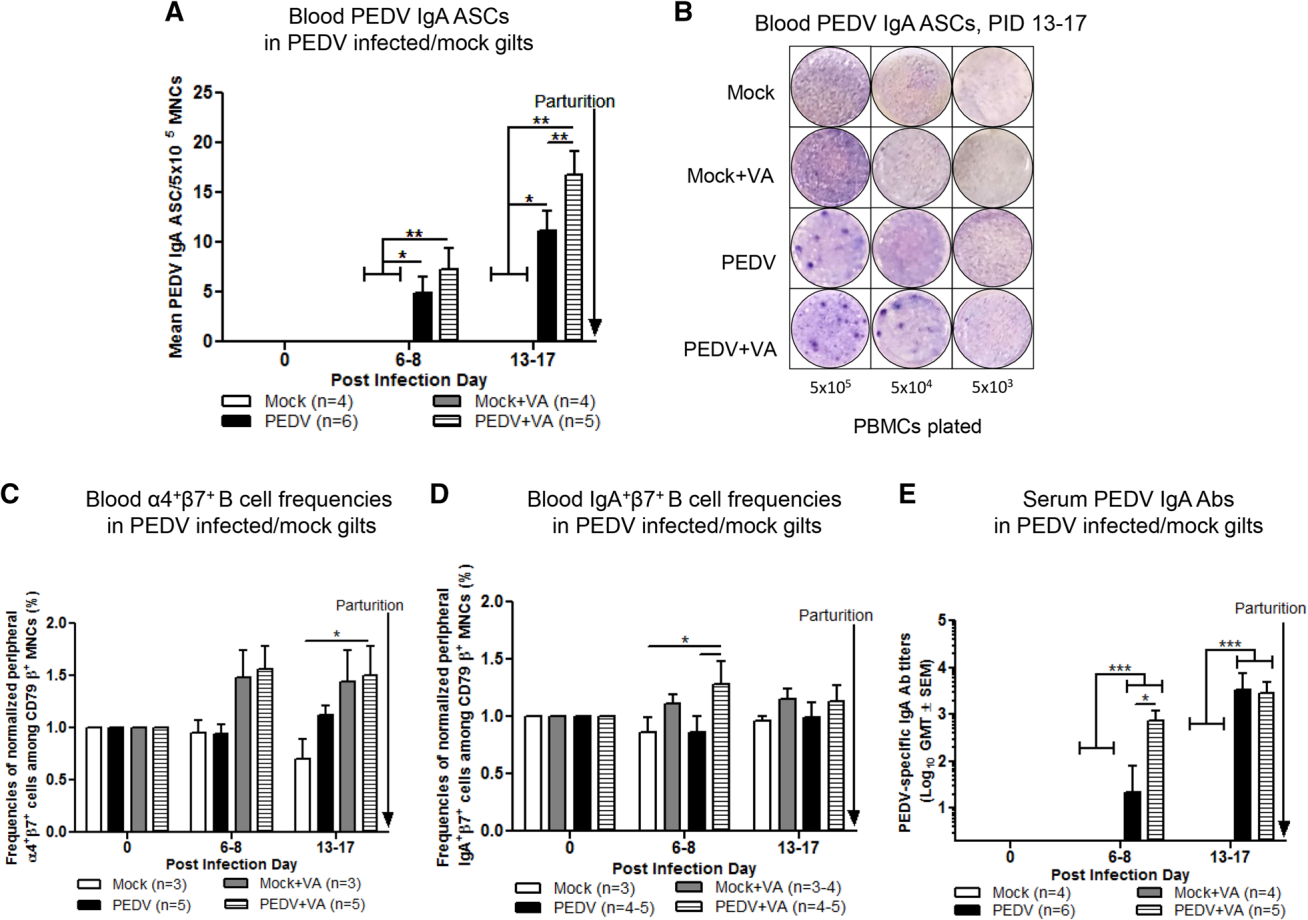
**Circulating PEDV specific IgA ASCs were significantly increased in PEDV + VA gilts pre-partum. A**, **B** Peripheral blood MNCs (PBMCs) were isolated and added to PEDV ELISPOT plates to determine the PEDV specific IgA ASCs. **C** PBMCs were isolated and analyzed for frequencies of α4^+^β7^+^ B cells and **D** IgA^+^β7^+^ MNCs by flow cytometry **E** PEDV IgA Abs were determined by ELISA. Asterisks indicate significant differences among treatment groups at the same time point (mean ± SEM). Frequencies of α4^+^β7^+^ B cells and IgA^+^β7^+^ MNCs were normalized by dividing by post-challenge day (PCD) 0 frequencies. Statistical analysis was performed using the two-way ANOVA with repeated measures and Bonferroni’s correction for multiple comparisons. *P < 0.05 **P < 0.01 ***P < 0.001.

The mean number of circulating PEDV IgA ASCs was significantly higher at PID 13–17 in PEDV + VA compared with PEDV gilts (Figures 3A and B). PEDV + VA gilts had significantly higher mean frequencies of circulating α4^+^β7^+^ B cells (normalized to PID 0) compared with mock gilts at PID 13–17 (Figure 3C). PEDV + VA gilts also had significantly higher mean frequencies of circulating IgA^+^β7^+^ MNCs (normalized to PID 0) at PID 6–8 compared with mock and PEDV gilts (Figure 3D). Whereas no significant differences were seen in pre-partum levels of circulating PEDV IgG ASCs, IgG Abs or VN Abs post PEDV infection (Additional file 2), PEDV IgA Ab titers were significantly higher at PID 6–8 in PEDV + VA compared with PEDV gilts (Figure 3E).

### PEDV + VA litters had decreased mortality and morbidity

**Figure 4:**
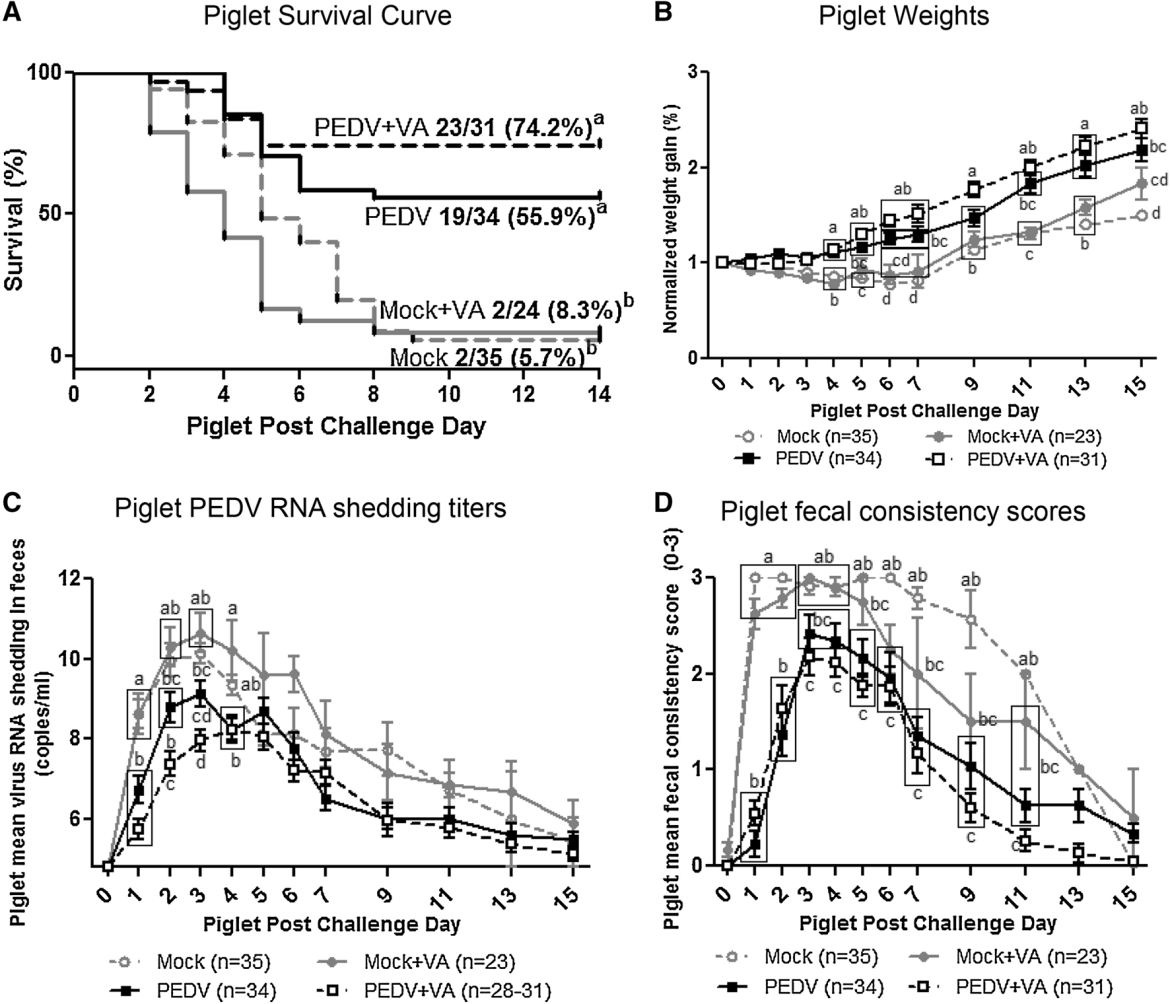
**PEDV + VA litters had increased survival rates and decreased PEDV RNA shedding titers. A** Kaplan–Meier survival curve of mock, mock + VA, PEDV and PEDV + VA litters at post-challenge day (PCD) 0–14. **B** Normalized weight gain of litters at PCD 0–15. Weights were normalized by dividing the daily weight (lbs) by PCD 0 weight (lbs). **C** Diarrhea was determined by fecal consistency score > 1 (fecal consistency was scored as follows: 0, normal; 1, pasty/semiliquid; 2, liquid; 3, watery). **D** Virus shedding was determined by real time quantitative polymerase chain reaction (RT-qPCR) and expressed as log_10_ copies/mL. Piglet diarrhea scores and viral shedding were measured at PCD 1–7, 9, 11, 13, and 15. Different alphabetical letters indicate significant differences among treatment groups at the same time point (mean ± SEM). Statistical analysis was performed using the log-rank (Mantel-Cox) test (**A**) or the two-way ANOVA with repeated measures and Bonferroni’s correction for multiple comparisons (**B**–**D**).

Piglets were PEDV-challenged at 3–5 days of age to determine the impact of maternal VA supplementation on lactogenic immunity and piglet morbidity and mortality. The survival rate of PEDV + VA litters was 74.2% compared with 55.9% in PEDV litters. Mock and mock + VA piglet survival rates were 5.7 and 8.3%, respectively (Figure 4A). Comparison of piglet weight gains revealed that PEDV + VA litters had significantly higher normalized weights at PCD 9 compared with PEDV litters (Figure 4B). Additionally, mock litters (± VA) were stunted (decreased or no weight gain) from PCD 1–7 and had the lowest normalized weights throughout the study. Although normalized mean weights at PID 15 were higher in mock + VA compared with mock piglets, these data represent only two surviving piglets in each respective treatment group. PEDV + VA piglets had significantly decreased PEDV RNA shedding titers at PCD 2 and lower and delayed mean peak shedding titers compared with PEDV piglets (Figure 4C). While not significantly different between piglets of PEDV + VA and PEDV gilts, fecal consistency scores decreased at a faster rate at PCD 7–15 in PEDV + VA compared with PEDV litters (Figure 4D). VA supplementation in mock gilts did not result in decreased morbidity or mortality in PEDV-challenged piglets.

### Oral VA supplementation increased milk PEDV IgA ASCs, total IgA IgSCs, and IgA^+^β7^+^ cells post piglet challenge

**Figure 5:**
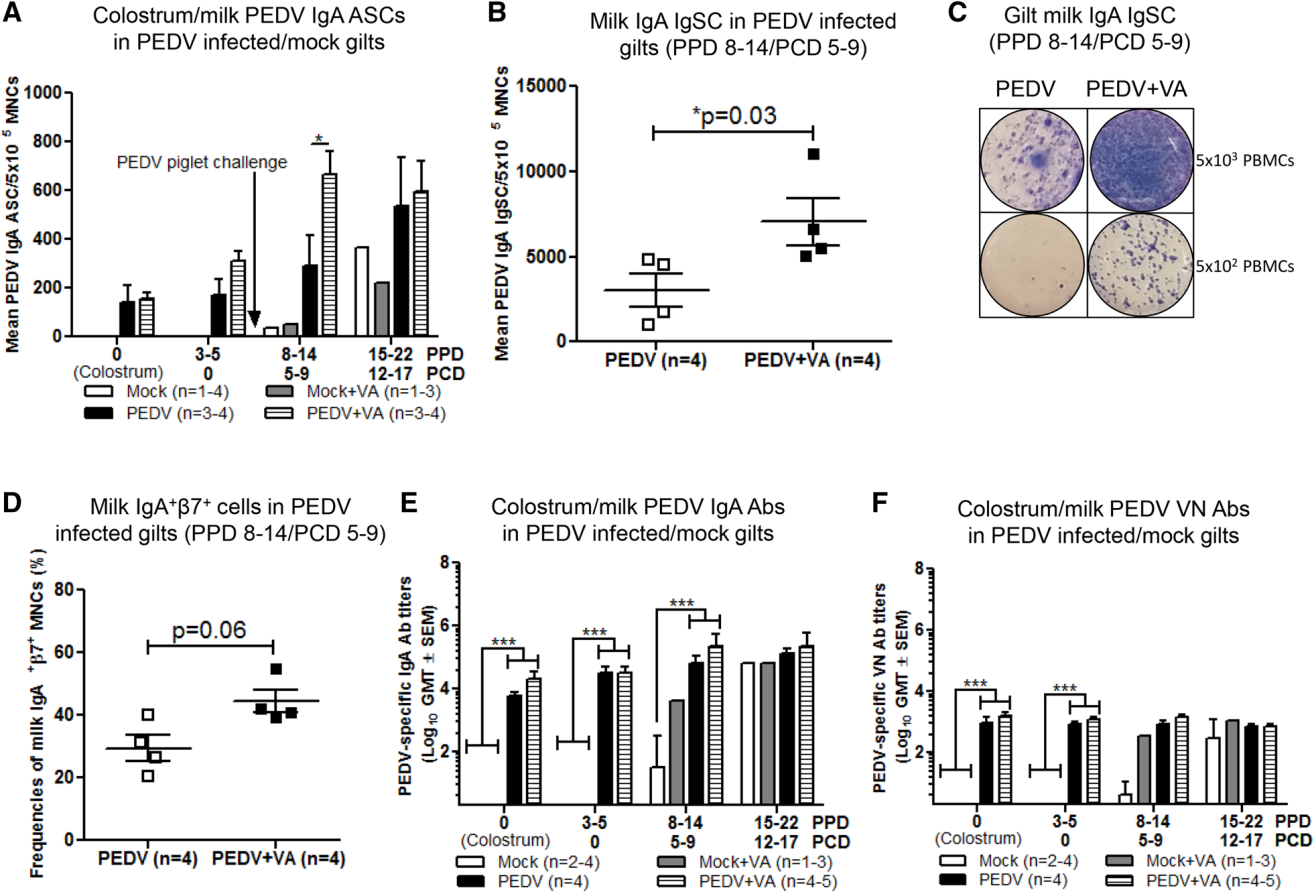
**PEDV + VA gilts had elevated levels of milk PEDV specific IgA ASCs and Abs, and IgA IgSCS. A** Milk MNCs were isolated and added to PEDV specific and **B**, **C** total ELISPOT plates to determine the PEDV specific IgA ASCs and IgA IgSCs. **D** Milk MNCs were isolated and analyzed for frequencies of IgA^+^β7^+^ B cells by flow cytometry **E** PEDV IgA and **F** virus neutralizing (VN) Abs were determined by VN Ab assay, respectively. Asterisks indicate significant differences among treatment groups at the same time point (mean ± SEM). Statistical analysis was performed using a Mann–Whitney t-test (**B**–**D**) or two-way ANOVA with repeated measures and Bonferroni’s correction for multiple comparisons (**A**, **E**, **F**). *P < 0.05 ***P < 0.001.

To determine the impact of maternal VA supplementation on lactogenic immunity, adaptive immune parameters were measured in colostrum and milk. Mean PEDV IgA ASCs in milk were significantly higher at PPD 8–14 (PCD 5–9) in PEDV + VA compared with PEDV gilts (Figure 5A). Additionally, mean milk IgA IgSCs at PPD 8–14 (PCD 5–9) were significantly higher in PEDV + VA compared with PEDV gilts at the same time point (Figures 5B and C). Milk MNCs from PEDV (± VA) gilts were isolated and stained for expression of IgA and mucosal homing receptor β7 integrin. Mean milk IgA^+^β7^+^ cell frequencies were numerically higher (*P* = 0.06) in PEDV + VA compared with PEDV gilts at PPD 8–14 (PCD 5–9) (Figure 5D). Due to the high mortality rate of mock (± VA) litters, the MGs of mock gilts regressed rapidly [42]. Therefore, there was not enough milk available from mock gilts to isolate MNCs at PPD 8–14 (PCD 5–9). Mean PEDV IgA and VN Ab titers were numerically higher [PPD 8–14 (PCD 5–9) for IgA Abs; PPD 3–5 (PCD 0) and PPD 8–14 (PCD 5–9) for PEDV VN Abs] in PEDV + VA compared with PEDV gilts and significantly higher in PEDV (± VA) compared with mock (± VA) gilts at similar time points (Figures 5E and F). This is consistent with our hypothesis that IgA ASC in milk may play a role in lactogenic immune protection in neonatal piglets against PEDV challenge.

### PEDV Ab^+^ cells in the MG increased post piglet PEDV challenge

**Figure 6:**
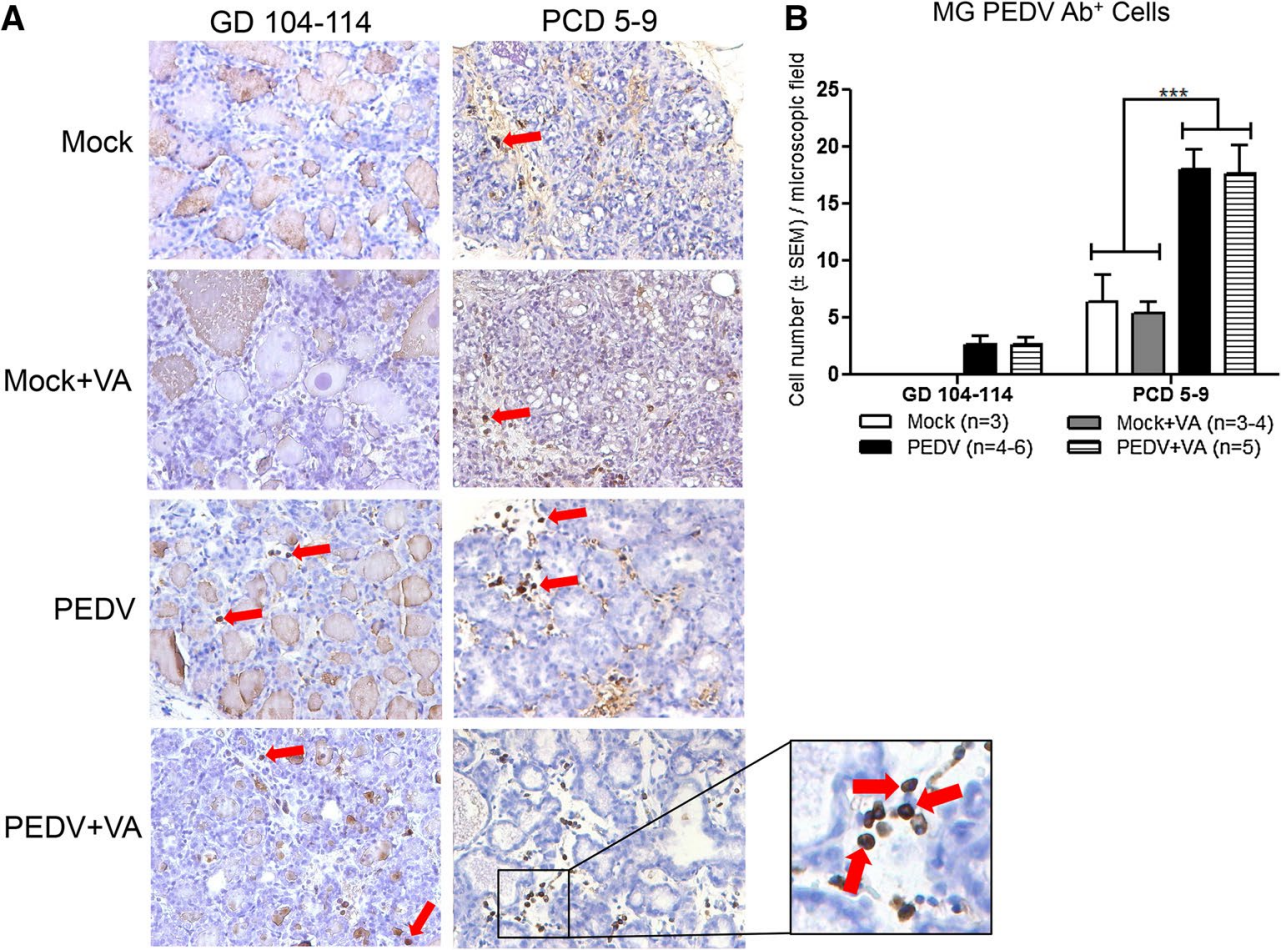
**PEDV antibody (Ab)**^**+**^ **cells in the mammary gland (MG) increased post PEDV challenge. A** Evaluation of anti-PEDV Ab^+^ plasma cells in the MG pre-partum [gestation day (GD) 104–114] and post-partum [post-challenge day (PCD) 5–9] in mock, mock + VA, PEDV and PEDV + VA gilts by PEDV viral suspension sandwich immunohistochemistry (IHC) method and hematoxylin staining (300×). Right: enlarged view of MG tissue where red arrows indicate PEDV Ab^+^ cells. **B** Cells were quantified by averaging PEDV Ab^+^ cells from 3 to 6 microscope fields (300×) from different areas of the MG from each animal within mock, mock + VA, PEDV and PEDV + VA gilts. Statistical analysis was performed using the one-way ANOVA. ***P < 0.001.

There were no differences in mean PEDV Ab^+^ cells per microscopic field at gilt GD 104–114 in PEDV + VA compared with PEDV gilts (Figures 6A and B). While there were no PEDV Ab^+^ cells in the MG of mock or mock + VA gilts at GD 104–114, PEDV + VA and PEDV gilts had significantly higher numbers of PEDV Ab^+^ cells per microscopic field (300×) in the MG at PCD 5–9 compared with mock gilts (± VA) (Figures 6A and B).

### PEDV exposure post piglet challenge affected circulating ASC and Ab responses in gilts and piglets

**Figure 7:**
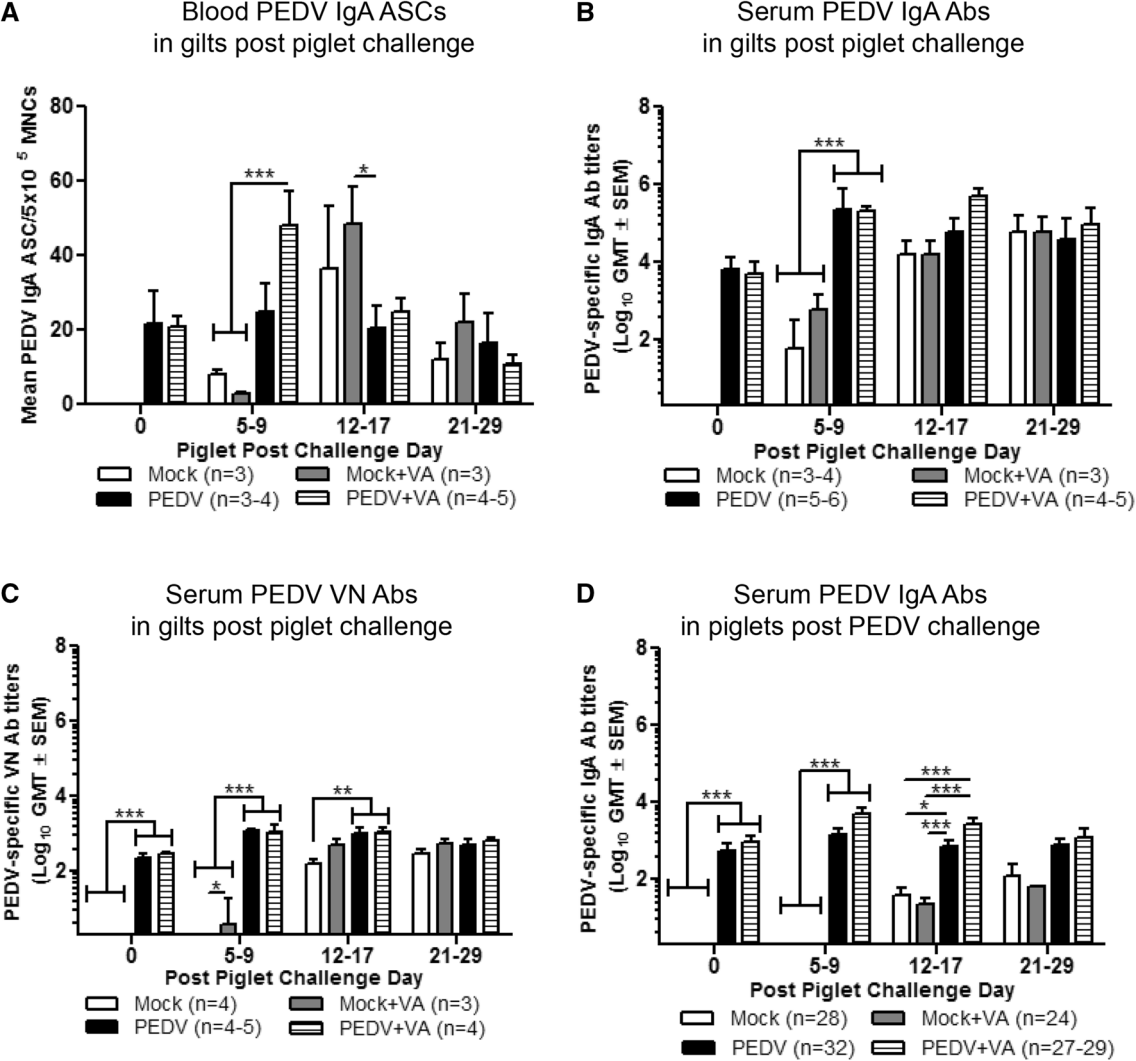
**Serum IgA antibodies (Abs) in PEDV (± VA) gilts increased to higher levels and at a faster rate than mock (± VA) gilts. A** Serum PEDV IgA antibody secreting cells (ASCs) and **B** Ab responses were determined by PEDV ELISPOT and ELISA assay, respectively. **C** Serum PEDV virus neutralizing (VN) Ab responses were determined by VN assay. **D** Piglet serum circulating PEDV IgA titers were measured by ELISA. Gilts and piglet serum was collected at piglet post-challenge day (PCD) 0, 5–9, 12–17 and 21–29. Asterisks indicate significant differences among treatment groups at the same time point (mean ± SEM). Statistical analysis was performed using the two-way ANOVA with repeated measures and Bonferroni’s correction for multiple comparisons. *P < 0.05 **P < 0.01 ***P < 0.001.

Circulating levels of PEDV IgA ASCs, PEDV IgA Abs and PEDV VN Abs were measured at PCD 0, 5–9, 12–17 and 21–29 in gilts and piglets. Circulating mean numbers of PEDV IgA ASCs in PEDV + VA gilts were significantly higher at PCD 5–9 compared with mock (± VA) gilts (Figure 7A). However, mock + VA gilts had significantly higher circulating PEDV IgA ASCs compared with PEDV gilts at PPD 12–17 (Figure 7A). Mean PEDV IgA Ab titers were numerically higher at PCD 12–17 and 21–29 in PEDV + VA compared with PEDV gilts (Figure 7B). PEDV gilts (± VA) had significantly elevated levels of PEDV IgA ASCs and Abs and VN Abs at PCD 5–9 compared with mock gilts (± VA) demonstrating an anamnestic response in PEDV gilts (± VA) (Figures 7A–C). Piglet mean PEDV IgA Ab titers were higher in PEDV + VA compared with PEDV (numerically) and mock (significantly) litters from PCD 0–17 (Figure 7D). Additionally, piglet serum mean PEDV IgA Ab titers increased in all treatment groups post piglet challenge (Figure 7D).

### IgA and IgG ASCs and ileal α4^+^β7^+^ B cells were increased in PEDV + VA gilts at PCD 21–29

**Figure 8:**
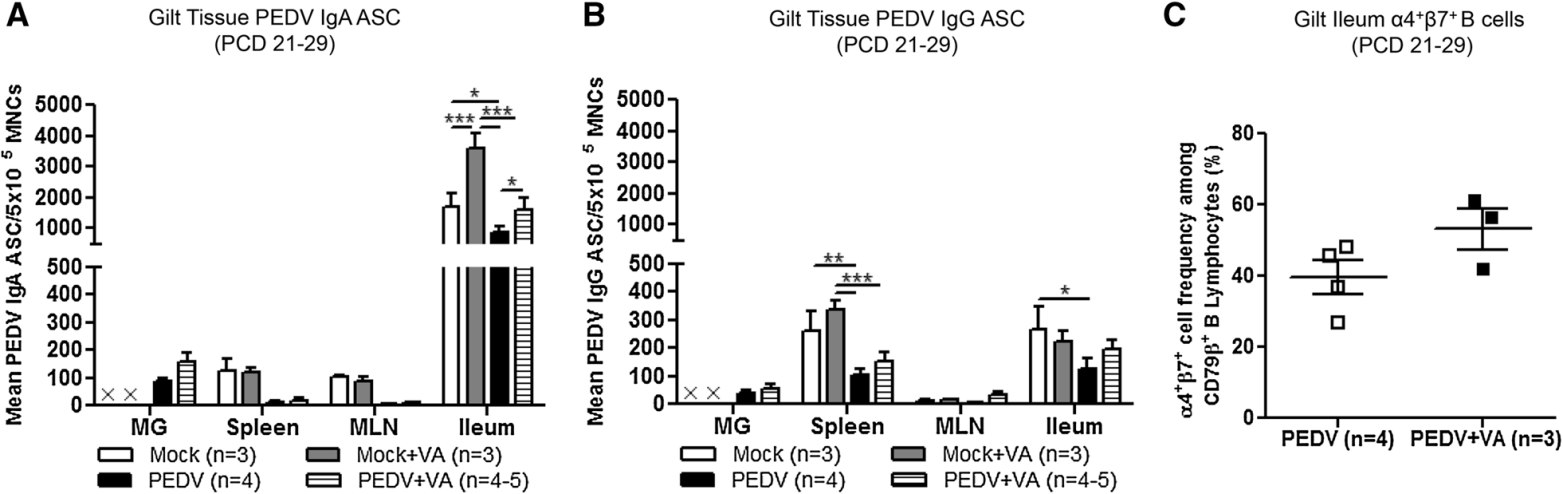
**IgA and IgG antibody secreting cells (ASCs) and α4**^**+**^**β7**^**+**^ **B cells in ileum increased in PEDV + VA gilts. A** Mammary gland (MG), spleen, mesenteric lymph node (MLN) and ileum mononuclear cells (MNCs) were isolated and added to PEDV ELISPOT plates to determine the PEDV specific IgA and **B** IgG ASCs. **C** Ileum MNCs were isolated and analyzed for frequencies of α4^+^β7^+^ B cells by flow cytometry. Asterisks indicate significant differences among treatment groups at the same time point (mean ± SEM). Statistical analysis was performed a two-way ANOVA with repeated measures and Bonferroni’s correction for multiple comparisons (**A**, **B**) or a Mann–Whitney t-test (**C**). *P < 0.05 **P < 0.01 ***P < 0.001.

Gilt MG, spleen, MLN and ileum tissues were analyzed for PEDV IgA and IgG ASCs at PCD 21**–**29. The mean numbers of IgA ASCs in spleen and MLN were numerically higher at PCD 21–29 in mock (± VA) compared with PEDV (± VA) gilts (Figure 8A). VA supplemented gilts in both mock and PEDV groups had significantly higher numbers of mean PEDV IgA ASCs in ileum compared with their respective nonsupplemented treatment group (Figure 8A). PEDV IgG ASCs in spleen were significantly higher in mock (± VA) compared with PEDV (± VA) gilts (Figure 8B). Additionally, mock gilts had significantly higher PEDV IgG ASCs in the ileum compared to PEDV gilts (Figure 8B). Due to the high mortality rate of mock (± VA) litters, MGs of mock gilts regressed rapidly [42]. Therefore, there was not enough MG tissue available to isolate MNCs and measure PEDV IgA and IgG ASCs. Additionally, mean frequencies of ileal α4^+^β7^+^ B cells were numerically higher in PEDV + VA compared with PEDV gilts (Figure 8C).

## Discussion

To our knowledge, this is the first study to evaluate the effect of VA supplementation on maternal and lactogenic immunity during an enteric viral infection in swine. Vitamin A is a critical mediator of mucosal immune function and is essential for lymphocyte trafficking in the gut. For example, VAD leads to increased susceptibility to bacterial and viral pathogens and VA supplementation alleviates the negative effects of certain infectious diseases [18, 27, 29, 43]. This is particularly relevant during pregnancy and the neonatal period when there is greater risk for clinical and subclinical VAD [44, 45]. To investigate the impact of VA on maternal and lactogenic immunity, we supplemented third trimester PEDV-infected gilts with oral retinyl acetate beginning at GD 76 and throughout lactation. We demonstrated that VA supplementation of PEDV-infected gilts decreased fecal consistency scores in third trimester gilts. Also, PEDV IgA ASCs and Abs, IgA IgSCs and IgA^+^β7^+^ B cells in blood and/or milk were greater in PEDV + VA gilts coinciding with greater mean survival rates in PEDV-challenged nursing piglets. Our findings suggest that daily oral VA supplementation during pregnancy may act as a mucosal adjuvant, enhancing maternal IgA and lactogenic immunity during PEDV infection.

Currently, VA recommendations in swine are 4000 IU/kg of feed (~7250 IU/day) during gestation and 2000 IU/kg of feed (fed ad libitum) during lactation [31]. However, these recommendations were developed assuming disease-free environments. Considering serum VA concentrations in swine decrease with increasing gestation [27] and parity (A.N. Vlasova and L.J. Saif, unpublished observations), it is likely that dietary VA recommendations for gestating gilts and sows are suboptimal during viral or bacterial infections. Due to the limited available studies on vitamin A supplementation in pregnant swine, we relied on literature from other species to inform our dose decision. Females of child-bearing age on a vitamin A-poor diet given a single oral dose of 30 000 IU retinyl palmitate produced a dose-related and sustained increase in plasma retinyl esters and retinoic acids [46]. Additionally, supplementing pregnant women with an oral dose of 10 000 IU/day of vitamin A resulted in enhanced prenatal H1N1-vaccine responses in mothers [47]. Therefore, if viral infection during swine pregnancy results in subclinical vitamin A deficiency, we reasoned that 30 000 IU would be an appropriate dose to elicit a physiological and immunological response. Additionally, based on nonhuman primate data, an oral dose of 30 000 IU retinoic acid is non-teratogenic [48]. Indeed, the presumed upper safe level of dietary VA is up to ten times the nutritional requirements in swine [31]. In in a previous study, harmful effects of VA toxicity were only observed in swine when more than 100 times the nutritional requirement was given over an extended period of time [31, 49]. Therefore, we reasoned that daily oral VA supplementation of 30 000 IU starting in the third trimester and lasting throughout lactation was unlikely to cause harmful effects. Indeed, no signs of hypervitaminosis A [50] were observed in gilts or piglets.

Serum retinol concentrations are homeostatically controlled over a range of adequate liver stores [51]; therefore it is not surprising that in our study, oral VA supplementation did not change serum retinol concentrations in gilts. Additionally, the rate of intestinal VA metabolism after oral supplementation contributes to the systemic exposure to VA metabolites [52]. For example, in feed-restricted lactating sows fed 35 µmol of 3,4-didehydroretinyl acetate (vitamin A2), serum 3,4-didehydroretinyl ester concentrations peaked 3 h post-prandial and returned to near baseline levels by 24 h [53]. If there were a transient increase in serum VA concentrations, it is unlikely that we would detect it as blood was collected from gilts immediately prior to VA supplementation at 0900 h. Similar to previous findings in swine and humans [27, 30], our study demonstrated decreased mean serum retinol concentrations immediately prior to parturition. This coincides at a time when chemokine CCR9 and CCR10 secretion and α4β7^+^ expression and their cognate receptors increase in the MG to recruit circulating ASCs into colostrum and milk [54, 55]. These results highlight the importance of understanding the role of VA during pregnancy and its potential effects on maternal immunity and the gut-MG-sIgA during an enteric viral infection.

We observed a lower and faster decline in mean peak PEDV RNA shedding titers and significantly decreased fecal consistency scores at PID 3 and 5 in PEDV + VA compared with PEDV gilts. This suggests that oral VA supplementation may enhance innate immune protection against PEDV in third trimester gilts. For example, previous studies of rats [56] and mice [57] reported decreased NK cell frequency and function during VAD and supplementation of β-carotene (a VA precursor) in vivo and in vitro increased NK cell cytotoxic activity [57]. This is in agreement with previous work in our lab demonstrating an association between increased NK cell frequencies in blood and decreased clinical signs in PEDV-challenged piglets [58]. PEDV infection is associated with local and systemic inflammation and increased concentrations of serum innate and proinflammatory cytokines [58, 59]. Indeed, protection against PEDV-induced disease was associated with a delay in serum proinflammatory cytokines post PEDV challenge in piglets [58]. Our findings demonstrate a trend whereby PEDV + VA gilts had numerically reduced mean proinflammatory cytokines and increased immunoregulatory and tissue repair cytokines IL-10 and IL-22, respectively, coinciding with significantly decreased diarrhea scores compared with PEDV gilts. These responses may aide in establishment of gut homeostasis, thereby reducing the severity of PEDV infection in third trimester gilts.

Oral VA supplementation significantly increased pre-partum levels of circulating PEDV IgA ASCs, IgA^+^β7^+^ MNCs and PEDV IgA Abs post PEDV infection. For example, the significant increase in circulating PEDV IgA Abs and ASCs at PID 6–8 and 13–17, respectively, coinciding with significantly increased normalized IgA^+^β7^+^ cells in PEDV + VA gilts, suggests that oral VA supplementation increased migration of PEDV stimulated B cells from the intestine into the circulation post PEDV infection. Indeed, trafficking IgA ASCs predominate in the lymphoid tissues of the gut [60] and migrate into the circulation after intestinal infection [39]. This is in agreement with previous studies from our lab demonstrating an IgA ASC dominant response after intestinal TGEV [61, 62], PEDV [16] and RV [39] infection in swine. Our lab also previously demonstrated that VA status influences anti-viral B cell immunity. For example, HRV-vaccinated VA sufficient piglets had increased HRV specific IgA ASCs and Abs in the duodenum post HRV challenge compared with vaccinated VAD piglets [29]. Furthermore, mucosal associated adhesion receptors are imprinted on immune cells responding to tissue-specific immune interactions [63]. For example, α4β7 integrin expression on intestinal lymphocytes increases after interaction with VA synthesizing DCs in intestinal Peyer’s patches, promoting cellular migration in the gut mucosa and lymphoid tissue [24]. In our study, mean frequencies of α4^+^β7^+^ B cells (normalized to PID 0) increased numerically in PEDV (± VA) gilts at PID 6–8 and 13–17 and mean frequencies of IgA^+^β7^+^ B cells were significantly higher at PID 13–17 in PEDV + VA compared with mock and PEDV gilts. This suggests that VA supplementation may influence migration of IgA^+^ cells after PEDV infection.

In our study, PEDV-challenged litters of PEDV + VA gilts had a higher survival rate, significantly decreased PEDV RNA shedding titers at PCD 2 and a trend for lower mean fecal consistency scores at PCD 7–15. The increased survival rates in PEDV + VA litters coincided with higher frequencies of circulating α4^+^β7^+^ B cells at PID 6–8 (normalized to PID 0) in gilts and IgA IgSC in milk at PPD 8–14 (PCD 5–9). These results are in agreement with previous work in our lab demonstrating that increased lactogenic immune protection in PEDV nursing piglets correlated with PEDV IgA ASCs and Abs in milk [16]. Additionally, in TGEV-infected sows, high rates of protection are associated with high titers of IgA Abs in colostrum and milk [8, 12, 64–66]. Similar findings were also reported in mice where supplementation of β-carotene (a proVA metabolite) during pregnancy and lactation increased IgA IgSC in the MG during lactation [67, 68]. The significant differences in milk PEDV IgA ASCs and total IgA IgSCs in PEDV + VA gilts were limited to post piglet PEDV challenge time points, suggesting a role for VA in enhancing anamnestic lactogenic immune responses. Future studies investigating the role of VA in milk and MG memory B cell function in swine are warranted.

While there were no differences in gilt serum immune responses between PEDV + VA and PEDV gilts post-partum, PEDV (± VA) gilt IgA ASCs and IgA and VN Abs increased to higher levels and at a faster rate compared with mock (± VA) gilts, suggesting an anamnestic response in PEDV-infected gilts after reexposure to PEDV and the naïve status of mock gilts at the time of piglet PEDV challenge. Lastly, gilt MG, spleen, MLN and ileum tissues were analyzed for PEDV IgA and IgG ASCs at PCD 21–29. Due to the high PEDV RNA shedding titers in the feces of mock litters, mock gilts had greater exposure to PEDV than PEDV (± VA) infected gilts during both primary (pre-partum) or secondary exposure (post piglet challenge). Therefore, the numbers of mean IgA ASCs in the spleen, MLN and ileum and IgG ASCs in the spleen and ileum were higher at PCD 21–29 in mock (± VA) compared with PEDV (± VA) gilts. Interestingly, however, VA supplemented gilts in both mock and PEDV groups had significantly higher mean PEDV IgA ASCs in the ileum compared with their respective nonsupplemented counterparts. Additionally, while not significant, mean frequencies of α4^+^β7^+^ B cells in ileum were higher in PEDV + VA compared with PEDV gilts. These data suggest that VA supplementation had an effect on local IgA immunity as demonstrated previously [24, 69] regardless of past PEDV exposure, potentiating the levels of trafficking IgA ASCs in serum and the MG. Considering that trafficking of IgA^+^ plasmablasts to the MG is dependent on CCR10 [70] and that RA influences CCR9 and α4β7^+^-dependent B cell homing in the intestine [23, 24], future investigation of these molecules and their cognate receptor expression and function in VAD models of swine pregnancy is warranted.

While there are indications that VA regulates maternal Ab responses to mucosal pathogens during pregnancy, its potential applications to promote the health of neonates remains unexplored. Using a pregnant swine model, our data demonstrate that VA supplementation in third trimester PEDV-infected gilts have a dual benefit to mother and neonate. VA supplementation resulted in less severe diarrhea and lower PEDV RNA shedding titers in pregnant gilts suggesting that VA supplementation promotes homeostasis and immune regulation in the gut post PEDV infection. Additionally, VA supplemented gilts had increased anti-PEDV IgA immunity in blood, milk and ileum. This was associated with increased survival rates in PEDV + VA litters post PEDV challenge. Based on our innovative approach and results demonstrating that VA enhanced intestinal immunity during pregnancy and lactogenic immune protection in nursing piglets, future studies are warranted to better understand the mechanisms involved. This model is applicable to endemic and emerging enteric viral diseases in humans and animals, as similar maternal vaccination and VA supplementation strategies may be needed to enhance the gut-MG-sIgA axis and neonatal protection.

## Supplementary information

**Additional file 1. Comparison of serum cytokine concentrations (A) interferon (IFN)-α, (B) IFN-γ, (C) interleukin (IL)-10, (D) IL-22, (E) tumor necrosis factor (TNF)-α (f) IL-6 (g) IL-12 (H) IL-4 and (I) transforming growth factor (TGF)-β in gilts at PID 0, 6–8 and 13–17.** Statistical analysis was performed using the two-way ANOVA with repeated measures and Bonferroni’s correction for multiple comparisons. Data are mean ± SEM.

**Additional file 2. Circulating levels of (A) PEDV IgG antibody secreting cells (ASCs), (B) PEDV IgG antibodies (Abs), (C) PEDV virus neutralizing (VN) Abs, (D) CD4**^**+**^ **T cells and (E) CD8**^**+**^ **T cells in mock, mock + VA, PEDV and PEDV + VA gilts at post-infection day (PID) 0, 6–8 and 13–17.** Asterisks indicate significant differences among treatment groups at the same time point (mean ± SEM). Statistical analysis was performed using the two-way ANOVA with repeated measures and Bonferroni’s correction for multiple comparisons. *P < 0.05 **P < 0.01 ***P < 0.001.

## Data Availability

The datasets generated and analyzed during the current study are available from the corresponding author on reasonable request.

## Abbreviations

sIgA: secretory IgA
Ab: antibody
PEDV: porcine epidemic diarrhea virus
MG: mammary gland
ASC: antibody secreting cell
RA: retinoic acid
VA: vitamin A
VAD: vitamin A deficient
LP: lamina propria
DC: dendritic cell
CCR: chemokine receptor
MLN: mesenteric lymph node
Th: T-helper cell
HRV: human rotavirus
IgSC: immunoglobulin secreting cell
VN: virus neutralization
TGEV: transmissible gastroenteritis virus
GD: gestation day
PID: post-infection day
MNC: mononuclear cells
PCD: post-challenge day
IM: intramuscular
PPD: post-partum day
RT qPCR: real-time quantitative polymerase chain reaction
TNF: tumor necrosis factor
IL: interleukin
IFN: interferon
TGF: transforming growth factor
GMT: geometric mean titer
ELISPOT: Enzyme-Linked Immunosorbent Spot

## References

[1] Kirk MD, Pires SM, Black RE, Caipo M, Crump JA, Devleesschauwer B, Döpfer D, Fazil A, Fischer-Walker CL, Hald T, Hall AJ, Keddy KH, Lake RJ, Lanata CF, Torgerson PR, Havelaar AH, Angulo FJ (2015). World Health Organization estimates of the global and regional disease burden of 22 foodborne bacterial, protozoal, and viral diseases, 2010: a data synthesis. PLoS Med.

[2] Saif LJ, Pensaert MB, Sestak K, Yeo SG, Jung K, Zimmerman JJ, Karriker LA, Ramirez A, Schwartz KJ, Stevenson GW (2012). Coronaviruses. Diseases of swine.

[3] Martella V, Banyai K, Matthijnssens J, Buonavoglia C, Ciarlet M (2010). Zoonotic aspects of rotaviruses. Vet Microbiol.

[4] Paarlberg PL (2014) Updated estimated economic welfare impacts of porcine epidemic diarrhea virus (PEDv). In: Working Paper #14-4. Purdue University

[5] Salmon H, Berri M, Gerdts V, Meurens F (2009). Humoral and cellular factors of maternal immunity in swine. Dev Comp Immunol.

[6] Ward LA, Rich ED, Besser TE (1996). Role of maternally derived circulating antibodies in protection of neonatal swine against porcine group A rotavirus. J Infect Dis.

[7] Bohl EH, Frederick T, Saif LJ (1975). Passive immunity in transmissible gastroenteritis of swine: intramuscular injection of pregnant swine with a modified live-virus vaccine. Am J Vet Res.

[8] Bohl EH, Gupta RK, Olquin MV, Saif LJ (1972). Antibody responses in serum, colostrum, and milk of swine after infection or vaccination with transmissible gastroenteritis virus. Infect Immun.

[9] Jayashree S, Bhan MK, Kumar R, Bhandari N, Sazawal S (1988). Protection against neonatal rotavirus infection by breast milk antibodies and trypsin inhibitors. J Med Virol.

[10] Saif LJ, Theil KW, Saif LJ, Heckert RA (1990). Enteric coronaviruses. Viral Diarrhea of Man and Animals.

[11] Olanratmanee EO, Kunavongkrit A, Tummaruk P (2010). Impact of porcine epidemic diarrhea virus infection at different periods of pregnancy on subsequent reproductive performance in gilts and sows. Anim Reprod Sci.

[12] Bohl EH, Saif LJ (1975). Passive immunity in transmissible gastroenteritis of swine: immunoglobulin characteristics of antibodies in milk after inoculating virus by different routes. Infect Immun.

[13] Langel SN, Paim FC, Lager KM, Vlasova AN, Saif LJ (2016). Lactogenic immunity and vaccines for porcine epidemic diarrhea virus (PEDV): historical and current concepts. Virus Res.

[14] Chattha KS, Roth JA, Saif LJ (2015). Strategies for design and application of enteric viral vaccines. Annu Rev Anim Biosci.

[15] Gerdts V, Zakhartchouk A (2017). Vaccines for porcine epidemic diarrhea virus and other swine coronaviruses. Vet Microbiol.

[16] Langel SN, Paim FC, Alhamo MA, Buckley A, Van Geelen A, Lager KM, Vlasova AN, Saif LJ (2019). Stage of gestation at porcine epidemic diarrhea virus infection of pregnant swine impacts maternal immunity and lactogenic immune protection of neonatal suckling piglets. Front Immunol.

[17] Brown CC, Noelle RJ (2015). Seeing through the dark: new insights into the immune regulatory functions of vitamin A. Eur J Immunol.

[18] Villamor E, Fawzi WW (2005). Effects of vitamin a supplementation on immune responses and correlation with clinical outcomes. Clin Microbiol Rev.

[19] Glasziou PP, Mackerras DE (1993). Vitamin A supplementation in infectious diseases: a meta-analysis. BMJ.

[20] Darlow BA, Graham PJ (2011). Vitamin A supplementation to prevent mortality and short- and long-term morbidity in very low birthweight infants. Cochrane Database Syst Rev.

[21] Coombes JL, Powrie F (2008). Dendritic cells in intestinal immune regulation. Nat Rev Immunol.

[22] Johansson-Lindbom B, Svensson M, Pabst O, Palmqvist C, Marquez G, Forster R, Agace WW (2005). Functional specialization of gut CD103+ dendritic cells in the regulation of tissue-selective T cell homing. J Exp Med.

[23] Mora JR, Iwata M, Eksteen B, Song SY, Junt T, Senman B, Otipoby KL, Yokota A, Takeuchi H, Ricciardi-Castagnoli P, Rajewsky K, Adams DH, von Andrian UH (2006). Generation of gut-homing IgA-secreting B cells by intestinal dendritic cells. Science.

[24] Iwata M, Hirakiyama A, Eshima Y, Kagechika H, Kato C, Song SY (2004). Retinoic acid imprints gut-homing specificity on T cells. Immunity.

[25] Wieringa FT, Dijkhuizen MA, West CE, van der Ven-Jongekrijg J, van der Meer JW (2004). Reduced production of immunoregulatory cytokines in vitamin A- and zinc-deficient Indonesian infants. Eur J Clin Nutr.

[26] Cantorna MT, Nashold FE, Hayes CE (1994). In vitamin A deficiency multiple mechanisms establish a regulatory T helper cell imbalance with excess Th1 and insufficient Th2 function. J Immunol.

[27] Vlasova AN, Chattha KS, Kandasamy S, Siegismund CS, Saif LJ (2013). Prenatally acquired vitamin A deficiency alters innate immune responses to human rotavirus in a gnotobiotic pig model. J Immunol.

[28] Chattha KS, Kandasamy S, Vlasova AN, Saif LJ (2013). Vitamin A deficiency impairs adaptive B and T cell responses to a prototype monovalent attenuated human rotavirus vaccine and virulent human rotavirus challenge in a gnotobiotic piglet model. PLoS One.

[29] Kandasamy S, Chattha KS, Vlasova AN, Saif LJ (2014). Prenatal vitamin A deficiency impairs adaptive immune responses to pentavalent rotavirus vaccine (RotaTeq(R)) in a neonatal gnotobiotic pig model. Vaccine.

[30] Radhika MS, Bhaskaram P, Balakrishna N, Ramalakshmi BA, Devi S, Kumar BS (2002). Effects of vitamin A deficiency during pregnancy on maternal and child health. BJOG.

[31] Council NR (2012). Nutrient requirements of swine: eleventh revised edition.

[32] Oka T, Saif LJ, Marthaler D, Esseili MA, Meulia T, Lin CM, Vlasova AN, Jung K, Zhang Y, Wang Q (2014). Cell culture isolation and sequence analysis of genetically diverse US porcine epidemic diarrhea virus strains including a novel strain with a large deletion in the spike gene. Vet Microbiol.

[33] Lin CM, Hou Y, Marthaler DG, Gao X, Liu X, Zheng L, Saif LJ, Wang Q (2017). Attenuation of an original US porcine epidemic diarrhea virus strain PC22A via serial cell culture passage. Vet Microbiol.

[34] Liu X, Lin CM, Annamalai T, Gao X, Lu Z, Esseili MA, Jung K, El-Tholoth M, Saif LJ, Wang Q (2015). Determination of the infectious titer and virulence of an original US porcine epidemic diarrhea virus PC22A strain. Vet Res.

[35] Jung K, Wang Q, Scheuer KA, Lu Z, Zhang Y, Saif LJ (2014). Pathology of US porcine epidemic diarrhea virus strain PC21A in gnotobiotic pigs. Emerg Infect Dis.

[36] Scheiermann C, Kunisaki Y, Frenette PS (2013). Circadian control of the immune system. Nat Rev Immunol.

[37] Pick R, He W, Chen CS, Scheiermann C (2019). Time-of-day-dependent trafficking and function of leukocyte subsets. Trends Immunol.

[38] Lin CM, Annamalai T, Liu X, Gao X, Lu Z, El-Tholoth M, Hu H, Saif LJ, Wang Q (2015). Experimental infection of a US spike-insertion deletion porcine epidemic diarrhea virus in conventional nursing piglets and cross-protection to the original US PEDV infection. Vet Res.

[39] Yuan L, Ward LA, Rosen BI, To TL, Saif LJ (1996). Systematic and intestinal antibody-secreting cell responses and correlates of protective immunity to human rotavirus in a gnotobiotic pig model of disease. J Virol.

[40] Sinkora M, Stepanova K, Sinkorova J (2013). Different anti-CD21 antibodies can be used to discriminate developmentally and functionally different subsets of B lymphocytes in circulation of pigs. Dev Comp Immunol.

[41] Pickert G, Neufert C, Leppkes M, Zheng Y, Wittkopf N, Warntjen M, Lehr HA, Hirth S, Weigmann B, Wirtz S, Ouyang W, Neurath MF, Becker C (2009). STAT3 links IL-22 signaling in intestinal epithelial cells to mucosal wound healing. J Exp Med.

[42] Kim SW, Easter RA, Hurley WL (2001). The regression of unsuckled mammary glands during lactation in sows: the influence of lactation stage, dietary nutrients, and litter size. J Anim Sci.

[43] de Medeiros P, Pinto DV, de Almeida JZ, Rego JMC, Rodrigues FAP, Lima AAM, Bolick DT, Guerrant RL, Oria RB (2018). Modulation of intestinal immune and barrier functions by vitamin A: implications for current understanding of malnutrition and enteric infections in children. Nutrients.

[44] Ahmed F, Prendiville N, Narayan A (2016). Micronutrient deficiencies among children and women in Bangladesh: progress and challenges. J Nutr Sci.

[45] West KP (2002). Extent of vitamin A deficiency among preschool children and women of reproductive age. J Nutr.

[46] Nohynek GJ, Meuling WJ, Vaes WH, Lawrence RS, Shapiro S, Schulte S, Steiling W, Bausch J, Gerber E, Sasa H, Nau H (2006). Repeated topical treatment, in contrast to single oral doses, with Vitamin A-containing preparations does not affect plasma concentrations of retinol, retinyl esters or retinoic acids in female subjects of child-bearing age. Toxicol Lett.

[47] Ahmad SM, Alam MJ, Khanam A, Rashid M, Islam S, Kabir Y, Raqib R, Steinhoff MC (2018). Vitamin A supplementation during pregnancy enhances pandemic H1N1 vaccine response in mothers, but enhancement of transplacental antibody transfer may depend on when mothers are vaccinated during pregnancy. J Nutr.

[48] Miller RK, Hendrickx AG, Mills JL, Hummler H, Wiegand UW (1998). Periconceptional vitamin A use: how much is teratogenic?. Reprod Toxicol.

[49] Anderson MD, Speer VC, McCall JT, Hays VW (1966). Hypervitaminosis A in the young pig. J Anim Sci.

[50] Pryor WJ, Seawright AA, McCosker PJ (1969). Hypervitaminosis A in the pig. Aust Vet J.

[51] Tanumihardjo SA (2004). Assessing vitamin A status: past, present and future. J Nutr.

[52] Arnhold T, Nau H, Meyer S, Rothkoetter HJ, Lampen AD (2002). Porcine intestinal metabolism of excess vitamin A differs following vitamin A supplementation and liver consumption. J Nutr.

[53] Surles RL, Li J, Tanumihardjo SA (2006). The modified-relative-dose-response values in serum and milk are positively correlated over time in lactating sows with adequate vitamin A status. J Nutr.

[54] Bourges D, Meurens F, Berri M, Chevaleyre C, Zanello G, Levast B, Melo S, Gerdts V, Salmon H (2008). New insights into the dual recruitment of IgA+ B cells in the developing mammary gland. Mol Immunol.

[55] Wilson HL, Obradovic MR (2015). Evidence for a common mucosal immune system in the pig. Mol Immunol.

[56] Bowman TA, Goonewardene IM, Pasatiempo AM, Ross AC, Taylor CE (1990). Vitamin A deficiency decreases natural killer cell activity and interferon production in rats. J Nutr.

[57] Ashfaq MK, Zuberi HS, Anwar Waqar M (2000). Vitamin E and beta-carotene affect natural killer cell function. Int J Food Sci Nutr.

[58] Annamalai T, Saif LJ, Lu Z, Jung K (2015). Age-dependent variation in innate immune responses to porcine epidemic diarrhea virus infection in suckling versus weaned pigs. Vet Immunol Immunopathol.

[59] Jung K, Miyazaki A, Saif LJ (2018). Immunohistochemical detection of the vomiting-inducing monoamine neurotransmitter serotonin and enterochromaffin cells in the intestines of conventional or gnotobiotic (Gn) pigs infected with porcine epidemic diarrhea virus (PEDV) and serum cytokine responses of Gn pigs to acute PEDV infection. Res Vet Sci.

[60] Kunkel EJ, Butcher EC (2003). Plasma-cell homing. Nat Rev Immunol.

[61] VanCott JL, Brim TA, Lunney JK, Saif LJ (1994). Contribution of antibody-secreting cells induced in mucosal lymphoid tissues of pigs inoculated with respiratory or enteric strains of coronavirus to immunity against enteric coronavirus challenge. J Immunol.

[62] VanCott JL, Brim TA, Simkins RA, Saif LJ (1993). Isotype-specific antibody-secreting cells to transmissible gastroenteritis virus and porcine respiratory coronavirus in gut- and bronchus-associated lymphoid tissues of suckling pigs. J Immunol.

[63] Seong Y, Lazarus NH, Sutherland L, Habtezion A, Abramson T, He XS, Greenberg HB, Butcher EC (2017). Trafficking receptor signatures define blood plasmablasts responding to tissue-specific immune challenge. JCI Insight.

[64] Saif LJ, Bohl EH, Gupta RK (1972). Isolation of porcine immunoglobulins and determination of the immunoglobulin classes of transmissible gastroenteritis viral antibodies. Infect Immun.

[65] Saif LJ (1999). Enteric viral infections of pigs and strategies for induction of mucosal immunity. Adv Vet Med.

[66] Moxley RA, Olson LD (1989). Clinical evaluation of transmissible gastroenteritis virus vaccines and vaccination procedures for inducing lactogenic immunity in sows. Am J Vet Res.

[67] Nishiyama Y, Sugimoto M, Ikeda S, Kume S (2011). Supplemental beta-carotene increases IgA-secreting cells in mammary gland and IgA transfer from milk to neonatal mice. Br J Nutr.

[68] Nishiyama Y, Yasumatsuya K, Kasai K, Sakase M, Nishino O, Akaike M, Nagase T, Sugimoto M, Ikeda S, Kume S (2011). Effects of supplemental β-carotene with whey on IgA transfer from maternal milk and mucosal IgA induction in neonatal mice and calves. Livest Sci.

[69] Stephensen CB, Moldoveanu Z, Gangopadhyay NN (1996). Vitamin A deficiency diminishes the salivary immunoglobulin A response and enhances the serum immunoglobulin G response to influenza A virus infection in BALB/c mice. J Nutr.

[70] Wilson E, Butcher EC (2004). CCL28 controls immunoglobulin (Ig)A plasma cell accumulation in the lactating mammary gland and IgA antibody transfer to the neonate. J Exp Med.

