# Supplementary figures and images for: Oral vitamin A supplementation of porcine epidemic diarrhea virus infected gilts enhances IgA and lactogenic immune protection of nursing piglets
Source: Vet Res. 2019 Nov 29;50:101. doi: 10.1186/s13567-019-0719-y (PMC6884901; doi:10.1186/s13567-019-0719-y)

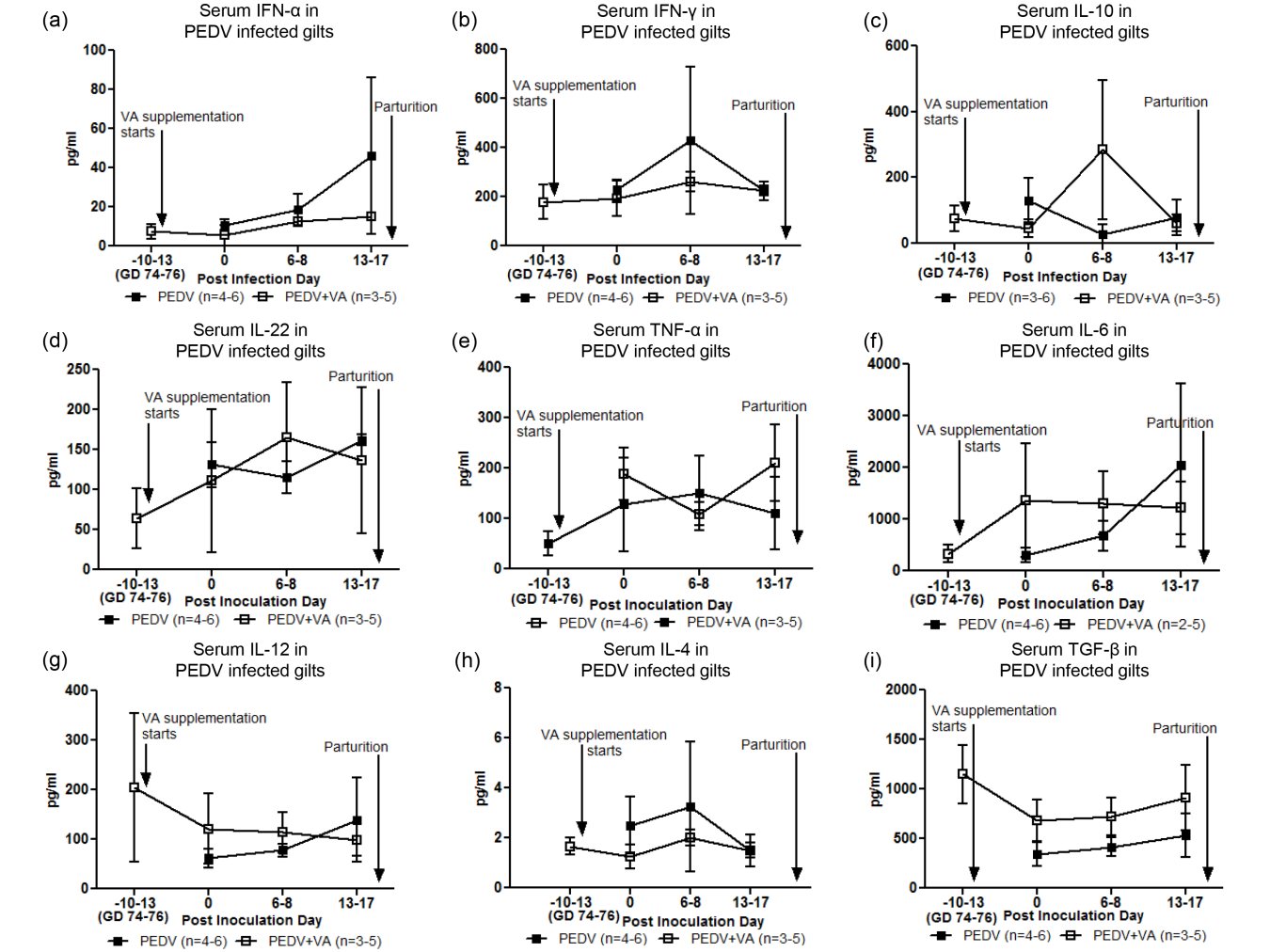


A

B

C

D

E

F

G

H

I

Supplement: Supplementary file 1 — Additional file 1. Comparison of serum cytokine concentrations (A) interferon (IFN)-α, (B) IFN-γ, (C) interleukin (IL)-10, (D) IL-22, (E) tumor necrosis factor (TNF)-α (f) IL-6 (g) IL-12 (H) IL-4 and (I) transforming growth factor (TGF)-β in gilts at PID 0, 6–8 and 13–17. Statistical analysis was performed using the two-way ANOVA with repeated measures and Bonferroni’s correction for multiple comparisons. Data are mean ± SEM. [file 13567_2019_719_MOESM1_ESM.docx]

**
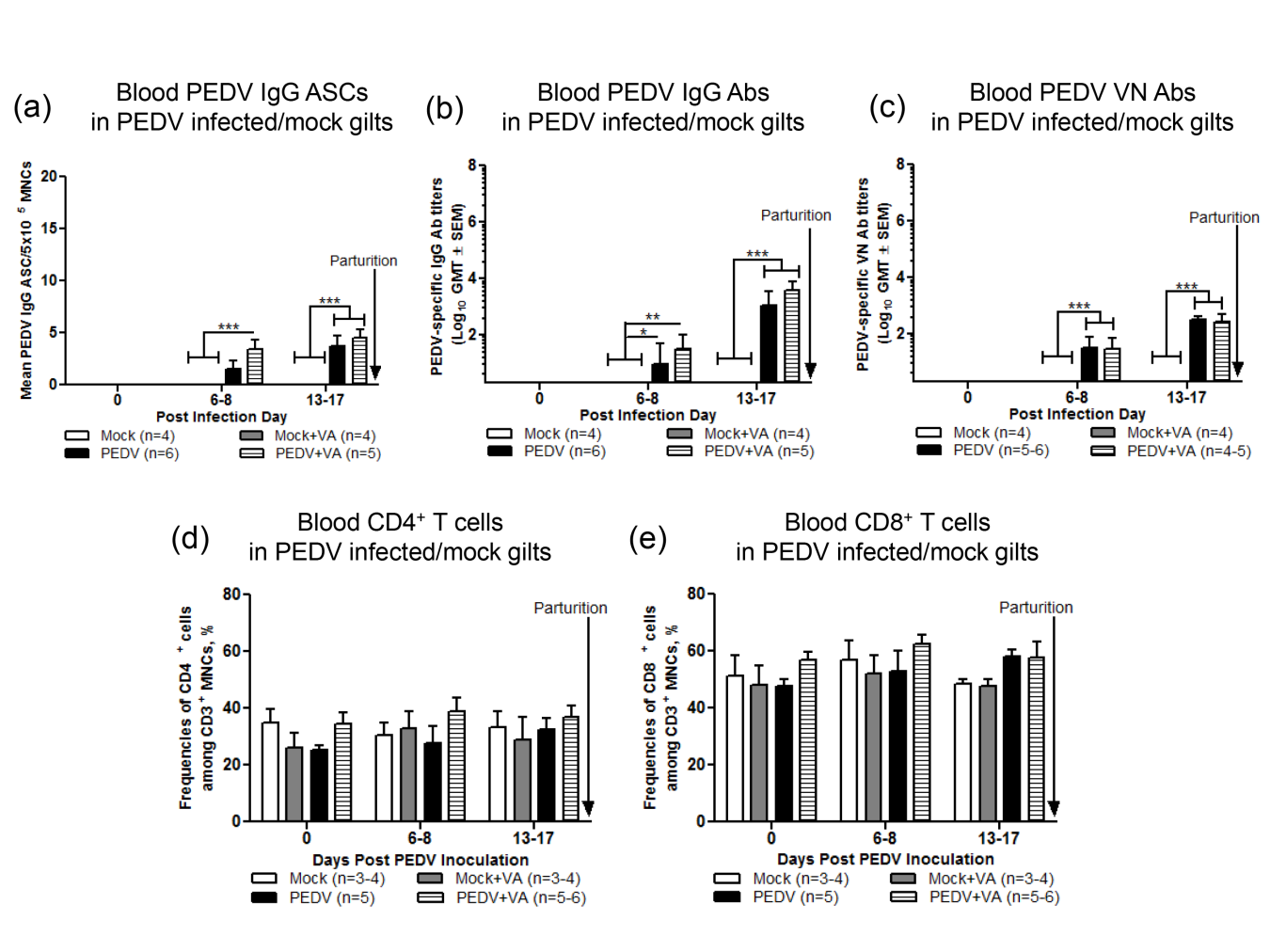
**


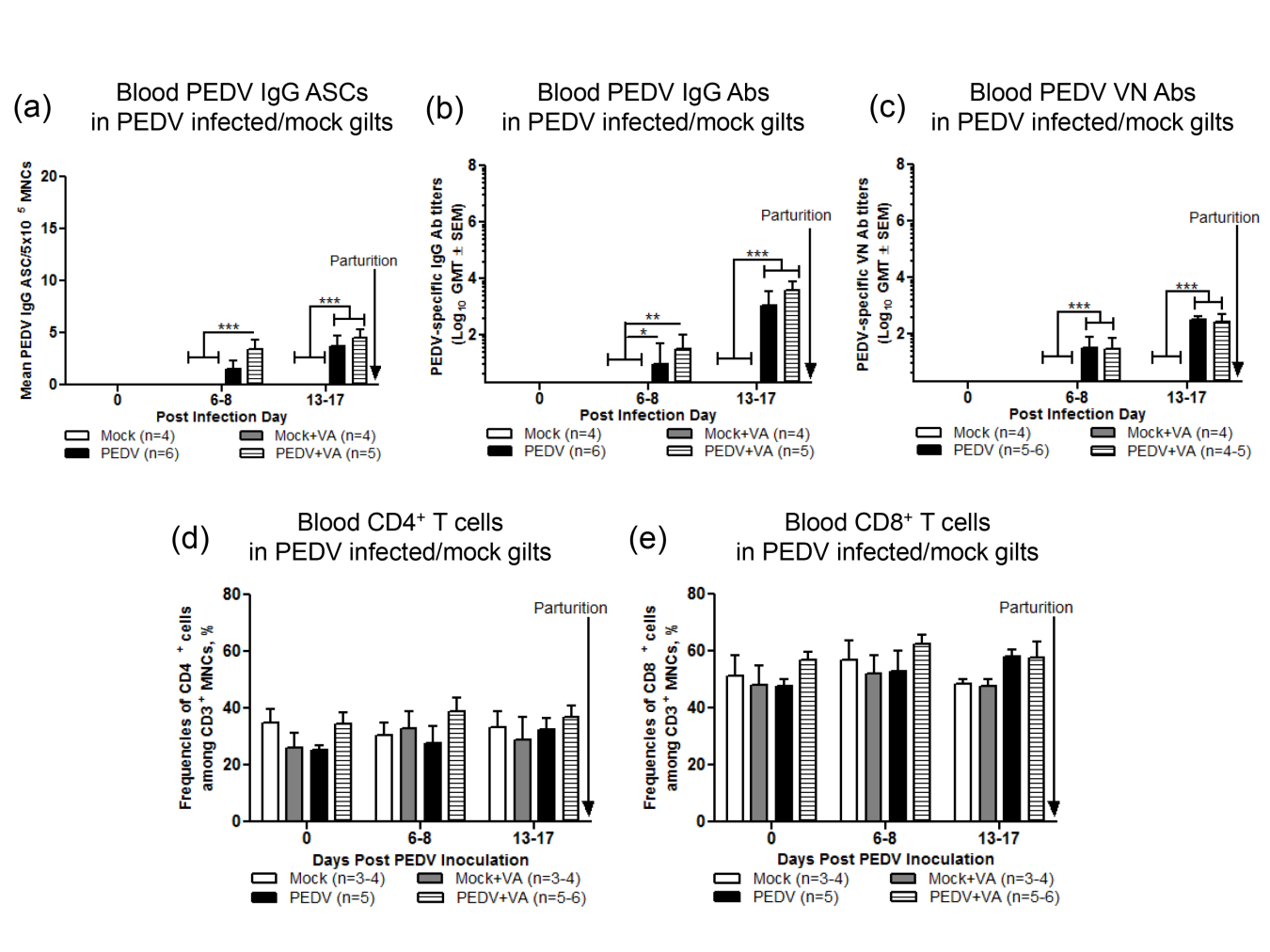


A

B

C

D

E

Supplement: Supplementary file 2 — Additional file 2. Circulating levels of (A) PEDV IgG antibody secreting cells (ASCs), (B) PEDV IgG antibodies (Abs), (C) PEDV virus neutralizing (VN) Abs, (D) CD4+ T cells and (E) CD8+ T cells in mock, mock + VA, PEDV and PEDV + VA gilts at post-infection day (PID) 0, 6–8 and 13–17. Asterisks indicate significant differences among treatment groups at the same time point (mean ± SEM). Statistical analysis was performed using the two-way ANOVA with repeated measures and Bonferroni’s correction for multiple comparisons. *P < 0.05 **P < 0.01 ***P < 0.001. [file 13567_2019_719_MOESM2_ESM.docx]
